# Research advances in improving drug efficacy through remodeling of tumor vascular function by different aerobic exercises

**DOI:** 10.3389/fspor.2026.1774978

**Published:** 2026-04-08

**Authors:** Shuaihao Hou, Rong Sun, Suqin Hu, Qing Zhang, Xiangdong Sun, Liqun Wang, Jianfeng Wu

**Affiliations:** 1 Department of Graduate School, Wannan Medical College, Wuhu, An Hui, China; 2 Department of Radiation Oncology, Jinling Hospital, Affiliated Hospital of Medical School, Nanjing University, Nanjing, China; 3 Department of Sick and Casualty Management, Nanjing Jinling Hospital, Affiliated Hospital of Medical School, Nanjing University, Nanjing, Jiangsu, China; 4 Department of Laboratory Medicine, Nanjing First Hospital, Nanjing Medical University, Nanjing, Jiangsu, China

**Keywords:** aerobic exercise, chemotherapy efficacy, drug delivery, exercise intensity, tumor microenvironment, tumor vasculature, vascular normalization

## Abstract

Structural and functional abnormalities of tumor vasculature are key factors limiting drug delivery and therapeutic efficacy. This review aims to systematically summarize the mechanisms and evidence regarding how aerobic exercise improves drug delivery and enhances tumor treatment response by remodeling tumor vascular function. By synthesizing recent studies, we summarize the effects of different aerobic exercise modalities (intensity, duration, frequency) on tumor vasculature and the synergistic effects of combining exercise with pharmacotherapy. Aerobic exercise can increase pericyte coverage and enhance vascular stability, reduce vascular permeability and tumor interstitial fluid pressure (IFP) to improve drug penetration, enhance tumor blood flow perfusion and oxygenation to alleviate the hypoxic microenvironment, and modulate tumor metabolism and acid-base balance. These effects collectively promote the distribution and accumulation of drugs within tumor tissue, thereby enhancing treatment efficacy. The effects of exercise are influenced by its intensity, duration, frequency, and tumor type, with moderate-intensity, regular regimens (e.g., 3–5 times per week) being the most substantiated. Aerobic exercise serves as an effective non-pharmacological adjuvant that can significantly improve drug delivery and enhance anti-tumor treatment outcomes through multi-dimensional remodeling of tumor vascular function and the tumor microenvironment. Future prospective clinical studies, incorporating imaging and molecular biomarkers, are needed to further define optimal exercise protocols and their role in individualized integrated cancer therapy.

## 1 Introduction

Malignant tumors represent a major global public health and economic burden, accounting for approximately one in six deaths worldwide and contributing to 30% of premature deaths from non-communicable diseases, establishing them as a primary obstacle to increasing life expectancy in this century (1). The high mortality associated with cancer is largely driven by malignant proliferation, metastasis, and poor prognosis. Emerging evidence highlights that microvascular dysfunction within the tumor microenvironment plays a critical role in these processes (2, 3). To support their uncontrolled growth, tumors must induce and sustain a dedicated blood supply, a process known as neovascularization. An imbalance between pro- and anti-angiogenic factors results in the formation of irregular tumor vessels characterized by disorganized endothelial cells and inadequate pericyte coverage. These structural abnormalities lead to increased vascular permeability, chaotic blood flow, and elevated interstitial fluid pressure, creating hypoxic and acidic regions within the tumor (4–7). This dysfunctional vasculature impedes the uniform delivery of drugs and oxygen, thereby promoting a more aggressive and therapy-resistant tumor phenotype (8). In clinical practice, achieving tumor vascular normalization has primarily relied on anti-angiogenic drugs; however, their efficacy is often transient and can be accompanied by significant side effects (9, 10). A growing body of evidence suggests that regular aerobic exercise can modulate the tumor microenvironment, particularly by remodeling its aberrant vasculature. Unlike pharmacological agents that directly target angiogenesis, aerobic exercise appears to enhance drug delivery and improve chemotherapy efficacy by restructuring tumor blood vessels (11). Studies in mouse models have demonstrated that exercise training can increase vascular maturity and perfusion, in part by enhancing pericyte coverage and reducing intratumoral hypoxia (12). These vascular changes are associated with improved drug delivery and tumor control; for instance, tumors in exercised animals exhibit deeper penetration of chemotherapeutic agents and slower growth compared to those in sedentary controls (13). Despite these promising findings, the precise molecular mechanisms by which exercise exerts these effects remain poorly understood. The Chinese guidelines for integrated tumor diagnosis and treatment recommend moderate-intensity aerobic exercise, defined as 40%–70% of VO_2_max or 50%–80% of HRmax (14). However, despite this clinical recommendation, key scientific questions remain unanswered. These include the optimal exercise “dose” (intensity, duration, and frequency), the dependency of its effects on specific tumor types, and how these parameters influence adaptive changes in the tumor vasculature.

**Figure 1:**
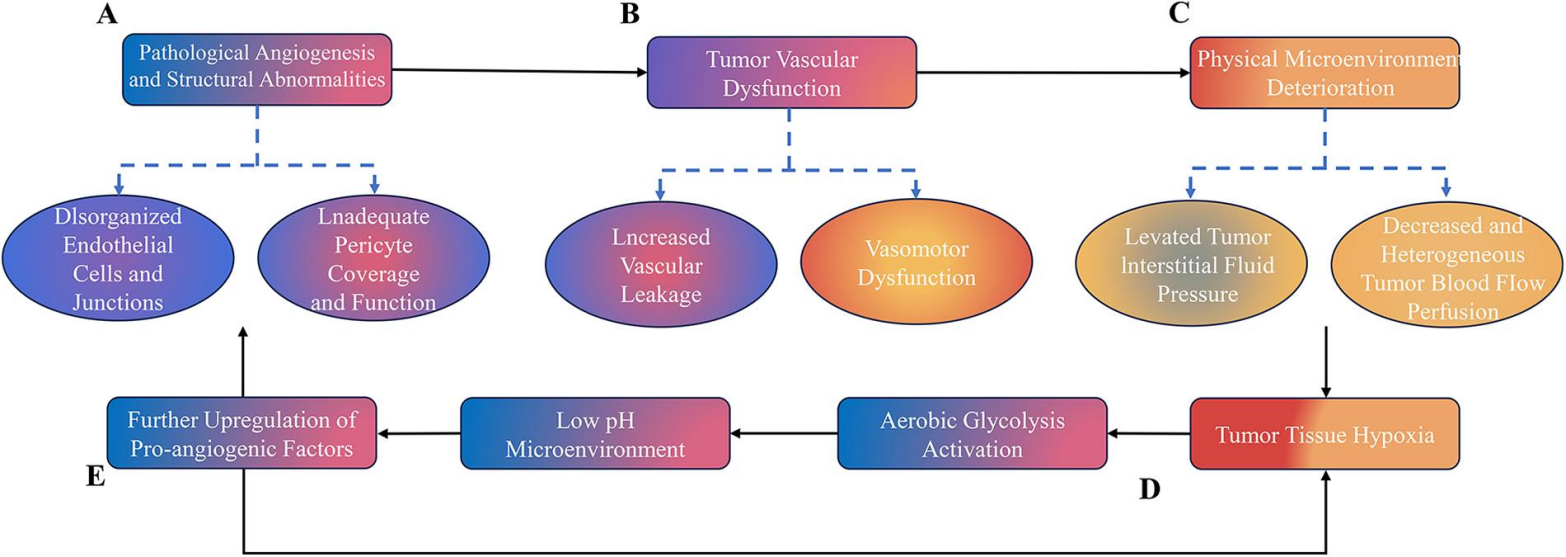
Impediment of tumor vascular abnormality vicious cycle to drug delivery (conceptual schematic). **(A)** Pathological Angiogenesis and Structural Abnormalities. The overexpression of pro-angiogenic factors, such as vascular endothelial growth factor (VEGF), drives pathological angiogenesis. This process results in newly formed blood vessels with significant structural abnormalities, including: ① disorganized endothelial cells and disrupted intercellular junctions; and ② inadequate pericyte coverage and functional defects. These structural deficits form the morphological basis for subsequent vascular dysfunction. **(B)** Vascular Dysfunction. The structural abnormalities described above directly lead to vascular dysfunction, characterized by: ① increased vascular permeability due to a compromised endothelial barrier; and ② impaired vasomotor function, as the loss of pericytes abolishes the vessels’ normal contractile regulation. Together, these functional abnormalities trigger critical downstream physical changes in the tumor microenvironment. **(C)** Physical Microenvironment Deterioration: The Primary Barrier to Drug Delivery. Vascular dysfunction directly induces adverse physical changes in the tumor microenvironment. These changes, primarily elevated interstitial fluid pressure and heterogeneous blood flow, constitute the most direct and significant obstacles to effective drug delivery. **(D)** Tumor Hypoxia and Acidosis. Insufficient and heterogeneous blood perfusion leads to tumor tissue hypoxia. Hypoxia stabilizes hypoxia-inducible factor 1-alpha (HIF-1*α*), which transcriptionally reprograms tumor cells toward aerobic glycolysis (the Warburg effect). This metabolic shift results in the excessive production of lactate, creating a low-pH microenvironment (acidosis). **(E)** Malignant Feedback Loops. Hypoxia, via HIF-1*α* signaling, upregulates pro-angiogenic factors such as VEGF, further exacerbating pathological angiogenesis and creating a self-amplifying loop. Concurrently, the acidic microenvironment compounds vascular dysfunction and promotes tumor invasion and immunosuppression.

This narrative review will focus on the relationship between aerobic exercise and tumor vascular function by addressing the following key aspects: First, the mechanisms and evidence by which aerobic training enhances pericyte coverage and vascular stability, reduces vascular permeability and interstitial fluid pressure, improves tumor blood perfusion and oxygenation, and helps correct tumor acidosis (Figure 1). Second, how different exercise modalities (varying in intensity, duration, and frequency) influence tumor vascular remodeling and the subsequent changes in the microenvironment. Finally, the mechanistic basis for the synergistic effects observed between aerobic exercise and drug therapy.

In summary, this narrative review aims to synthesize current evidence and discuss aerobic exercise as a translational strategy for remodeling tumor vasculature and improving drug delivery. By highlighting how exercise-induced vascular normalization can enhance the delivery and efficacy of chemotherapeutic agents, this review underscores the potential of aerobic exercise as a promising non-pharmacological adjunct in oncology.

The synergistic effect of these interconnected pathological alterations leads to a marked reduction in drug delivery efficiency. This is manifested by the inadequate penetration and heterogeneous distribution of chemotherapeutic agents within the tumor, ultimately compromising antitumor efficacy. Solid arrows denote linear cause-and-effect relationships driving the core pathological progression. Dashed arrows represent the specific biological phenotypes and mechanistic components that emanate from upstream pathological nodes.

## 2 Aerobic exercise enhances the efficacy of chemotherapy

### 2.1 Remodeling of the tumor vascular system by aerobic exercise

The tumor vascular network is a key determinant of the hostile tumor microenvironment, influencing various aspects including tumor perfusion, oxygenation, interstitial fluid pressure (IFP), pH, and immune responses (15). Unlike physiological angiogenesis, tumor vasculature develops severe structural and functional abnormalities. It is often dilated and tortuous, lacks a hierarchical branching pattern, and may exhibit fenestrated vessel walls with discontinuous or absent basement membranes and reduced pericyte coverage (16). This dysfunctional vasculature leads to blood extravasation into the interstitial space, which impairs normal perfusion and elevates IFP (17). Further research has demonstrated that anti-angiogenic therapy can reverse tumor neovascularization and normalize tumor vasculature, giving rise to the concept of tumor vascular normalization (18). This concept, initially proposed by Jain and colleagues, posits that restoring the balance of angiogenic regulators by reducing pro-angiogenic factors can remodel tumor vasculature, enhance its function, and render it more akin to normal vascular systems (19). Given that most pharmacological strategies for tumor vascular normalization are limited by side effects and transient efficacy, non-pharmacological approaches represent an attractive alternative. Emerging evidence indicates that regular physical exercise positively influences vascular remodeling, offering a promising non-pharmacological strategy for improving tumor vasculature.

#### 2.1.1 Improving pericyte coverage and vessel maturity

Exercise promotes the structural maturation of tumor vasculature, with increased pericyte coverage serving as the structural foundation for functional normalization. Mature vessels consist of quiescent endothelial cells surrounded by a complete layer of pericytes. Research by Betof et al. (12) revealed the impact of exercise training on tumor vascular structure. In breast cancer mouse models receiving chemotherapy, exercised mice showed a 3–4 fold higher co-localization level of the endothelial cell marker CD31 and the pericyte marker desmin compared to sedentary controls. The proportion of vessels wrapped by pericytes and the area of vessel coverage by pericytes within viable tumor regions were also significantly increased. This structural optimization indicates enhanced vascular stability. The underlying molecular mechanism involves exercise-induced shear stress, which triggers calcium influx into endothelial cells, activating calcineurin. This leads to the dephosphorylation and nuclear translocation of the transcription factor NFAT (Nuclear Factor of Activated T-cells). Nuclear NFAT then upregulates the expression of thrombospondin-1 (TSP-1). As a key signaling molecule, TSP-1 mediates communication between shear-stressed endothelial cells and surrounding cells, inhibiting excessive proliferation of primitive endothelial cells, promoting vessel differentiation, stabilization, and maturation, thereby reducing non-functional sprouting vessels and achieving more complete pericyte coverage (20, 21). Vessel maturation directly provides the foundation for improved function. Exercise-induced increases in pericyte coverage result in more stable and functionally competent vessels. Gomes-Santos et al. (22) observed in a breast cancer mouse model that exercise training delayed tumor growth and induced vascular normalization, manifested as increased pericyte coverage and perfusion along with reduced hypoxia. Furthermore, this exercise-improved vascular microenvironment subsequently reprogrammed the immune tumor microenvironment (TME) and enhanced CD8+T cell-mediated anti-tumor activity via CXCR3 signaling, improving responses to immunotherapy. This demonstrates that exercise enhances tumor treatment sensitivity multi-dimensionally through both vascular normalization and optimization of the immune microenvironment.

#### 2.1.2 Reducing vascular permeability and tumor interstitial fluid pressure

Abnormal tumor vessels, due to incomplete endothelial junctions, defective basement membranes, and insufficient pericyte coverage, exhibit high permeability (23). The mechanism primarily involves the excessive secretion of permeability factors such as vascular endothelial growth factor (VEGF), chemokine CCL2, and fibrinogen by tumor cells and inflammatory cells within the TME. These factors directly bind to endothelial junction molecules like VE-cadherin and Claudin-5, or activate signaling pathways such as Src and RhoA, inducing phosphorylation and degradation of junctional proteins. This disrupts the integrity of tight and adherens junctions between endothelial cells, leading to a hyper-leaky vascular state (24). In tumors, the incomplete or even absent endothelium and discontinuous basement membrane increase vascular permeability, causing blood extravasation into the interstitial space. Coupled with dysfunctional lymphatic drainage within tumors, this leads to interstitial fluid accumulation and a significant elevation of tumor interstitial fluid pressure (IFP) (25, 26). Elevated IFP constitutes a malignant physical barrier to treatment. This interstitial hypertension can cause edema and, due to erythrocyte stasis, lead to blood flow stagnation and subsequent vessel collapse. This collapse not only limits oxygen supply to the tumor, creating a vicious cycle (27), but also restricts the perfusion and delivery of chemotherapeutic drugs, reducing treatment efficacy (28). Moreover, the mechanical stress from high IFP itself can directly promote tumor progression. van der Voort van Zyp et al. (29) found that an increase of just 15 mmHg in pressure could stimulate colon cancer cell adhesion, leading to tumor recurrence. Aerobic exercise can effectively ameliorate this pathological state. Morrell et al. (11) found that two weeks of moderate-intensity exercise in a Ewing sarcoma mouse model significantly reduced tumor vascular hyperpermeability. The study showed that S1PR1 expression is associated with reduced vascular permeability, while S1PR2 expression is linked to pathological endothelium and higher permeability. Two weeks of treadmill exercise increased S1PR1 signaling and decreased S1PR2 signaling in tumor-bearing mice, thereby lowering tumor vascular permeability. Furthermore, exercise promotes lymphatic return through muscle fiber contraction during the muscle pump action, enhances skeletal muscle blood flow, and inhibits interstitial fluid accumulation by reducing post-capillary resistance, thereby comprehensively preventing IFP elevation (17).

#### 2.1.3 Increasing tumor blood flow perfusion

Normal vessels are lined by a monolayer of interconnected, adherent endothelial cells aligned with the direction of blood flow for optimal perfusion (30). Blood flow perfusion within tumor tissue is often disordered and insufficient. The irregular vascular network, presence of arteriovenous shunts, and lack of effective regulatory mechanisms prevent matching the metabolic demands of the tissue. These abnormal structures increase vascular resistance and blood viscosity, collectively hindering the effective delivery of oxygen, nutrients, and chemotherapeutic drugs to the tumor parenchyma (31–33). Therefore, improving tumor blood flow perfusion is a key step for enhancing drug delivery efficiency and alleviating hypoxia. Research indicates that during exercise, increased cardiac output and systemic blood pressure, combined with the inability of tumor vessels (which lack smooth muscle) to effectively contract, leads to a substantial drop in vascular resistance. This results in increased intratumoral blood flow, ultimately improving tumor perfusion levels (34, 35). McCullough et al. (36) found that acute exercise could increase the number of perfused vessels in prostate tumors by 200% and reduce hypoxic areas by 50%. Even a single bout of acute exercise can have a significant synergistic effect with chemotherapy. Multiple studies have shown that exercise combined with chemotherapy inhibits tumor growth more effectively than chemotherapy alone, a mechanism closely related to improved tumor blood perfusion and promoted drug delivery (11, 37). It is noteworthy that exercise-induced improvements in perfusion and reductions in hypoxia do not always occur simultaneously and may be modulated by factors such as tumor location and exercise intensity. For instance, Buss et al. (38) found that exercise could reduce tumor hypoxia without significantly altering tumor perfusion or vascular density, suggesting the existence of metabolic regulatory mechanisms beyond perfusion.

#### 2.1.4 Alleviating tumor hypoxia

Tumor hypoxia is not merely a static state of insufficient oxygen supply; it is a key driver of cancer malignancy and is considered an important target in cancer therapy (39). Tissue hypoxia stabilizes hypoxia-inducible factors (HIFs). In locally advanced solid tumors, prevalent hypoxic regions stabilize the HIF-α subunit, activating downstream gene expression that promotes abnormal angiogenesis, epithelial-mesenchymal transition, and metabolic reprogramming (e.g., the Warburg effect), thereby creating a vicious cycle that maintains tumor invasion, metastasis, and therapy resistance (40). Aerobic exercise provides a unique non-pharmacological avenue with the potential to enhance tumor oxygenation and possibly alleviate the hypoxic microenvironment associated with conventional anticancer therapy failure and aggressive tumor phenotypes. Exercise can directly and rapidly improve tumor oxygenation. McCullough et al. (36) studied the acute effects of exercise training on tumor hypoxia and confirmed that a single bout of treadmill running reduced the tumor hypoxic fraction by 15%. Long-term aerobic exercise intervention has cumulative effects, showing better outcomes compared to single-session interventions. Xiao et al. (41) observed that swimming exercise intervention in nude mice with subcutaneous hepatocellular carcinoma xenografts effectively alleviated intratumoral hypoxia. More importantly, this hypoxia alleviation is built upon deep vascular and metabolic remodeling. Aerobic exercise can increase tumor vascular density, improve vessel maturity, and enhance tumor perfusion and oxygen delivery, further reducing hypoxic areas. The mechanism may be related to exercise-mediated regulation of tumor metabolic intermediate levels and inhibition of HIF-1*α* stabilization (42). It should be noted that HIF reduction is an important indicator, but tumor alleviation depends not only on the transient inhibition of this single signaling node, but more on the persistent, benign remodeling of the microenvironment involving multiple elements such as vascular structure, immune infiltration, and the host's systemic state.

#### 2.1.5 Regulating acid-base balance

The aberrant metabolism of tumors is the root cause of their acidic microenvironment. Driven by factors like hypoxia, tumor cells tend to perform high-rate glycolysis even under aerobic conditions, consuming large amounts of glucose and producing excess lactate (43). Lactate, the end product of anaerobic glycolysis, is directly generated from pyruvate reduction catalyzed by lactate dehydrogenase (LDH) and is a hallmark of the Warburg phenotype. Excess lactate produced by tumor cells is extruded across the cell membrane via monocarboxylate transporters (especially MCT1 and MCT4), lowering the interstitial pH and creating a uniquely acidic microenvironment (44, 45). This acidic microenvironment adversely affects treatment and prognosis. Low pH has been shown to reduce cellular radiosensitivity and modulate the cytotoxicity of certain anticancer drugs (46). Furthermore, the acidic microenvironment promotes tumor cell survival, proliferation, and anti-apoptosis, induces immunosuppression, and further exacerbates malignant progression (47). Research indicates that aerobic exercise can modulate the acid-base balance in tumors. Aveseh et al. (13) reported that treadmill exercise in breast cancer-bearing mice reduced tumor and circulating lactate concentrations by approximately 17% compared to sedentary mice. Correspondingly, Bacurau et al. (48) found that a moderate-intensity treadmill exercise protocol significantly reduced glucose consumption and lactate production in Walker-256 tumor-bearing rats. One hypothesis suggests that physical exercise induces transient systemic acidosis. This periodic, brief decrease in blood pH may further lower the pH of the intratumoral microenvironment, thereby inducing cancer cell death and delaying or blocking the evolution of cancer cells toward malignant, invasive phenotypes (49).

### 2.2 Improved tumor vascular function enhances chemotherapeutic drug delivery

Effective pharmacotherapy relies on sufficient and uniform distribution of drugs within tumor tissue (17). Drug delivery in solid tumors faces multiple barriers. Tumor cells proliferate faster than capillary endothelial cells, forcing tumor vessels apart and forming a network unfavorable for drug delivery. Tumor cells are often located about 100 micrometers from the nearest vessel, whereas cells in normal tissues are closer to capillaries (50–100 μm). Drug molecules therefore need to diffuse over longer distances, making it difficult for most anticancer drugs to penetrate tumor cells (50–52). Research suggests that exercise training may also improve the penetration of chemotherapeutic drugs into tumors. Recent studies indicate that aerobic exercise training, by promoting vascular normalization, may serve as a non-pharmacological strategy to overcome these delivery bottlenecks. Schadler et al. (53) found that exercise alone did not slow tumor growth, but the combination of exercise and chemotherapy significantly enhanced the anti-tumor effect. Further analysis showed higher expression of DNA damage markers in tumor cells from the exercise-combination group, suggesting that more chemotherapeutic drugs successfully reached and exerted their effects. Alves et al. (54) found that moderate-intensity aerobic exercise improved chemotherapy outcomes in mice inoculated with lung cancer cells. Compared to doxorubicin treatment alone, tumor regrowth speed after chemotherapy cessation was slowed, and premature death due to doxorubicin toxicity was reduced. These results collectively indicate that exercise can substantially improve the delivery and intratumoral effect of chemotherapeutic drugs. At the structural level, direct evidence for vascular normalization comes from clinical research. Florez Bedoya et al. (55) conducted a study on 70 patients with potentially resectable pancreatic cancer who performed ≥120 min of moderate-intensity exercise weekly preoperatively. They found a doubling of the total number of vessels, significantly higher microvascular density, longer and more open vessel lumens, and vascular function closer to normalization. In summary, aerobic exercise does not directly kill tumors but remodels tumor vasculature, making its function and structure tend toward normal. These data suggest that exercise may improve tumor blood flow perfusion, thereby facilitating the smooth delivery of chemotherapeutic agents to the tumor target sites, leading to better chemotherapy efficacy. Exercise intervention can serve as an effective adjuvant to chemotherapy regimens.

## 3 Research on different aerobic exercises in cancer patients

### 3.1 Exercise intensity

Exercise intensity is a key parameter affecting the intervention effect of exercise on tumor tissue microvascular function. Considering the special condition of cancer patients and safety factors, moderate-intensity aerobic exercise protocols are commonly used in clinical settings. The Chinese Technical Guidelines for Integrated Cancer Diagnosis and Treatment recommend low-to-moderate intensity aerobic exercise at 40%–70% VO_2_max or 50%–80% HRmax (14). The advantage of moderate-intensity exercise lies in providing appropriate stimulation, stably increasing cardiac output and reasonably redistributing blood flow. It generates suitable fluid shear stress, activating vascular homeostatic pathways (e.g., NFAT-TSP-1/S1PR1) while avoiding endothelial damage. Moreover, the moderate sympathetic arousal it induces, leveraging the inherent *α*-adrenergic contractile deficiency of tumor vessels, redistributes blood flow to tumor regions, achieving a balance between improved perfusion and vascular protection (11).

At the basic research level, Schadler et al. (53) demonstrated that moderate-intensity exercise, by increasing shear stress and activating the calcineurin-NFAT-TSP-1 pathway in endothelial cells, significantly increased pericyte coverage on tumor vessels and upregulated *α*-smooth muscle actin expression, directly promoting capillary structural maturation. At the clinical translation and validation level, Bedoya et al. (14) conducted a home-based moderate-intensity exercise program for pancreatic cancer patients and found that the tumor vasculature was remodeled: the total number of vessels per tumor region doubled, with higher microvascular density, longer vessels, and more open lumens. Similarly, Van Blarigan et al. (56) examined whether physical activity was associated with tumor vessel morphology in men with prostate cancer. They found that men with moderate activity levels had larger and more regularly shaped vessels compared to those with low activity levels.

In recent years, multiple studies have shown that both moderate-intensity continuous exercise (MICE) and high-intensity interval exercise (HIIE) positively affect lung cancer treatment and can delay tumor progression. However, evidence regarding the safety and efficacy of high-intensity interval training (HIIT) in cancer patients and survivors remains relatively novel. Compared to healthy individuals, cancer patients have significantly reduced physical function and exercise tolerance. Therefore, high-intensity exercise loads may cause hypoxia and adversely affect tumor tissue microvascular function. Research by Gomes-Santos et al. (57) provides direct evidence for the advantage of moderate intensity. They found that when mice were exercised at low, moderate, or high intensity based on their individual maximum running capacity, only moderate-intensity exercise delayed tumor growth and reduced tumor burden, while low and high-intensity exercise failed to exert similar anti-tumor effects. Although both low and high-intensity exercise normalized tumor vasculature, only moderate-intensity exercise increased tumor-infiltrating CD8+ T cells, which also exhibited enhanced effector function. This suggests that the optimal exercise intensity may exist within a “window” that simultaneously optimizes vascular remodeling and immune modulation. Another experimental study on lung cancer mice found that compared to high-intensity interval exercise, moderate-intensity continuous exercise significantly inhibited lung tumor growth. MICE also reduced MMP9 levels in lung cancer tissue, potentially controlling tumor metastasis to some extent (58).

In summary, most existing interventional studies have employed moderate-intensity aerobic exercise. However, it remains unclear whether a true intensity–response relationship exists—for instance, whether higher exercise intensities confer proportionally greater improvements in tumor perfusion—or whether the benefits plateau beyond a certain threshold. Regarding the application of high-intensity interval training (HIIT) in oncology, a critical distinction must be made between systemic health outcomes and local tumor vascular effects. Furthermore, the systemic health benefits of HIIT in cancer patients are also a subject of debate. Meta-analytic evidence indicates that HIIT confers meaningful systemic benefits in cancer patients and survivors, notably by improving cardiorespiratory fitness (e.g., increasing VO_2_max) (59). Whether HIIT can effectively induce tumor vascular normalization or alleviate intratumoral hypoxia, however, remains to be established. Some preclinical studies have even suggested that high-intensity exercise may exert uncertain effects on the tumor microenvironment, potentially due to increased systemic hypoxic stress (60). Future research should prioritize well-designed mechanistic studies that incorporate functional imaging modalities-such as dynamic contrast-enhanced MRI and PET-to directly assess the impact of HIIT on local tumor perfusion, hypoxia, and vascular function, rather than relying solely on surrogate measures of systemic cardiorespiratory fitness.

### 3.2 Exercise timing

The effects of exercise on tumor vasculature can be distinguished as acute (single bout) effects and chronic (long-term) adaptations based on the duration of intervention. Different exercise types share the same direction of action, with improving tumor vascular function as the core.

Acute vascular adaptation refers to the immediate regulation of tumor blood flow by a single exercise bout. Gouez et al. (61) demonstrated that acute exercise prior to immunochemotherapy significantly reduced early tumor growth in a mouse model. McCullough et al. (36) injected tumor cells into the ventral prostate of rats and found that the maximum contraction elicited by norepinephrine in tumor arterioles was attenuated by approximately 95%. A 5-minute low-intensity treadmill running experiment showed a significant increase in the number of patent tumor vessels, a 200% increase in blood flow perfusion, a significant rise in oxygen partial pressure, and a 50% reduction in tumor tissue hypoxia. However, this acute improvement is not universal. Elming et al. (62) conducted 30 min of aerobic exercise on breast tumor-bearing mice and found that running reduced the tumor tissue hypoxic fraction by 37%, but a similar effect was not observed in a model with dorsal subcutaneous tumors. This discrepancy may be related to the vascular reactivity characteristics of the tumor location. Exercise activates the sympathetic nervous system, releasing norepinephrine. However, the *α*-adrenergic contractile response of tumor arterioles is blunted, and myogenic tone is lost under high intraluminal pressure, weakening vasoconstrictive regulation of blood flow. Additionally, subcutaneous adipose tissue (the host tissue for metastatic tumors) inherently has less blood flow. During exercise, blood is redistributed, prioritizing muscle perfusion, while skin blood flow may be reduced, ultimately leading to decreased blood flow to tumor tissue (62, 63). This indicates that the manifestation of acute effects depends on the local vascular biology of the tumor.

Chronic vascular adaptation stems from structural and functional remodeling of tumor vasculature induced by long-term, regular exercise training. Long-term exercise induces sustained structural and molecular adaptations, including enhanced endothelial stability, increased pericyte coverage, and improved remodeling of both angiogenesis and arteriogenesis (17, 53). Chronic, regular aerobic exercise exposes the vasculature to repeated shear stress stimuli, which progressively reinforce vascular normalization and enhance perfusion efficiency. This process ultimately leads to structural and functional adaptations within the tumor vasculature, primarily reflected in markedly improved tumor oxygenation and perfusion (64). In the same study by McCullough et al. (65) using a prostate cancer rat model, after 5–7 weeks of treadmill exercise training, the oxygen partial pressure (PO₂) in tumor microvessels increased by approximately 100%, and the hypoxic tumor area decreased dramatically from 39% to 4%. In a liver cancer model, 4 weeks of swimming exercise not only alleviated hypoxia but also suppressed HIF-1*α* and its downstream pro-survival signaling pathways (41). The core mechanism lies in the sustained shear stress from regular exercise, which activates the calcineurin-NFAT pathway in endothelial cells, upregulating key proteins like TSP-1, thereby driving vessel maturation and stability, ultimately achieving lasting improvements in perfusion and oxygenation (37).

However, exercise-induced tumor vascular adaptation does not consistently appear under all experimental conditions. Buss et al. (38) found in breast cancer and melanoma mouse models over approximately 3 weeks that while exercise induced physiological adaptations in peripheral organs like the heart, it did not systematically improve tumor growth, hypoxia, perfusion, or vascular density. This negative result suggests that factors such as exercise duration, timing of intervention, or the inherent biological characteristics of the tumor may be critical variables determining whether the vascular normalization effect can be achieved. Furthermore, Niemiro et al. (66) demonstrated that 8–12 weeks of moderate-intensity aerobic exercise was more significant in activating systemic and tumor-local anti-tumor immunity (e.g., promoting CD8+ effector T cell function, reducing T cell exhaustion). In the process of vascular normalization, aerobic exercise can also improve the immune microenvironment, with both synergistically promoting improved tumor treatment efficacy. Veras et al. (67) subjected 20 male rats with chemically induced prostate tumors to 8 weeks of exercise (moderate intensity, 5 times per week). They found that aerobic physical exercise significantly reduced prostate cell proliferation, tumor size, and prostate weight. It also significantly decreased anti-apoptotic protein expression and increased pro-apoptotic protein expression.

### 3.3 Exercise frequency

The World Health Organization's Guidelines on Physical Activity and Sedentary Behavior recommend that adults engage in aerobic exercise 3–5 times per week (68). Currently, there is a lack of clinical trials directly comparing the impact of different frequencies on key cancer treatment endpoints. In current research, protocols of 3–5 sessions per week are the mainstream choice. These protocols essentially represent a “regular, frequent” exercise pattern, which, by repeatedly applying hemodynamic stimuli, continuously drives adaptive signaling in vascular endothelial cells, thereby stabilizing vascular structure. Too low a frequency (e.g., ≤1 session per week) may be insufficient to form an effective cumulative stimulus, resulting in weak or non-persistent vascular adaptive responses. Dethlefsen et al. (69) conducted a 6-month exercise intervention (once weekly) in breast cancer survivors and found that post-training resting serum did not alter cancer cell viability. Conversely, an excessively high frequency, especially without corresponding adjustments in intensity or duration, may increase overall fatigue, inflammatory responses, or overtraining risk, potentially offsetting the benefits of exercise or being detrimental to cancer patients with already compromised immune function (70).

### 3.4 Synergy between exercise and therapy

The heterogeneous blood flow perfusion, interstitial hypertension, and hypoxic microenvironment caused by the abnormal internal vascular network of tumors constitute serious physical barriers to drug penetration and uniform distribution. Regular aerobic exercise, however, can precisely address this through multiple mechanisms such as inducing vascular structural maturation, reducing vascular permeability, increasing perfusion, and alleviating hypoxia. The synergy between exercise and therapy is not a simple additive effect but reflects the complementary role of exercise in enhancing treatment. Studies by Schadler et al. (53) and Alves et al. (54) both suggest that exercise combined with chemotherapy can significantly increase intratumoral drug exposure and damage markers. Amin et al. (71) combined exercise intervention with pharmacotherapy in breast tumor-bearing mice. They found that 5 weeks of intermittent aerobic exercise combined with drug therapy inhibited VEGF expression in breast tumor tissue via the miR-21, miR-206, and let-7a pathways, suppressed tumor microvascular generation and growth, and the effect of the combined intervention was superior to exercise alone. Zylstra et al. (72) introduced exercise prehabilitation during neoadjuvant therapy for esophageal cancer. The exercise group showed significantly enhanced tumor treatment response, and the median T-lymphocyte count was also significantly higher than in the control group. In summary, aerobic exercise systematically reshapes tumor vascular function and the immune microenvironment. On this foundation, combined pharmacotherapy creates a more favorable context for treatment efficacy. Future research could explore optimal exercise combination modes based on different tumor types and treatment regimens.

## 4 Conclusion

Based on the accumulating evidence, there is growing clinical interest in incorporating exercise as an adjunctive strategy to modulate tumor vascular function and enhance cancer treatment outcomes. These emerging data provide a rationale for integrating exercise into oncology practice. This review synthesizes evidence demonstrating that aerobic exercise-particularly at moderate intensity-can serve as an effective non-pharmacological intervention. By remodeling tumor vasculature and function through multiple mechanisms (e.g., enhancing pericyte coverage, improving perfusion, and alleviating hypoxia), exercise facilitates drug delivery and potentiates therapeutic efficacy. From a practical perspective, moderate-intensity aerobic training has demonstrated favorable safety, tolerability, and broad physiological benefits in cancer populations. Although such regimens have not yet been optimized specifically for modulating tumor perfusion, their underlying mechanisms-such as increased shear stress and enhanced nitric oxide bioavailability-overlap with cardiovascular training responses, supporting their biological plausibility for vascular effects.

Although preclinical studies have demonstrated that exercise training influences blood flow and subsequent tumor outcomes, clinical investigations assessing these effects in cancer patients remain scarce. Jones et al. (72) compared aerobic training combined with doxorubicin-cyclophosphamide neoadjuvant chemotherapy versus chemotherapy alone on breast tumor blood flow. However, due to limited sample size, the study was underpowered to accurately isolate the independent effect of exercise on tumor perfusion. This limitation underscores the need for further in-depth research on exercise in oncology, particularly regarding its effects on tumor vasculature. Most mechanistic insights are derived from animal models, and high-quality clinical studies directly demonstrating exercise-induced tumor vascular normalization-and quantitatively linking these changes to therapeutic outcomes-remain limited. While these models are valuable, they do not fully recapitulate the complexity and inter-individual variability of human tumor biology. Furthermore, heterogeneity in study designs—including variability in cancer types and stages-limits the generalizability of findings. These gaps hinder our ability to draw definitive conclusions and underscore the need for rigorous, mechanistically informed studies to establish the clinical relevance of exercise-induced tumor adaptations.

Clinical translation warrants caution. Tumor vascular responses are likely to vary by cancer type, as different tumor types or subtypes exhibit marked differences in microvascular density, angiogenic signaling (e.g., VEGF overexpression), and baseline perfusion levels. Moreover, more direct evidence is needed regarding the safety and efficacy of high-intensity interval training (HIIT) in cancer patients and survivors. While some studies have reported benefits of high-intensity training on VO₂max in cancer patients, they lack direct measurements of tumor hypoxia or oxygenation. It remains unclear whether higher exercise intensities confer proportionally greater improvements in tumor perfusion or whether a ceiling effect exists. Future trials should compare different exercise intensities-such as moderate versus high-intensity training-to determine the optimal “dose” for inducing vascular changes.

Although human evidence remains limited, preliminary data suggest that exercise may influence tumor biology, including vascular remodeling and treatment response. A deeper understanding of how exercise affects tumor vasculature could inform novel therapeutic strategies that complement conventional cancer treatments, ultimately improving patient outcomes and enabling more personalized treatment approaches. These insights also point toward future research directions. Well-designed prospective clinical trials are needed to directly validate these findings in humans, incorporating advanced imaging modalities (e.g., dynamic contrast-enhanced MRI, PET, Doppler ultrasound) and pathological assessments. Mechanistic studies should focus on solid tumors with high vascular dependence (e.g., breast, pancreatic, and renal cancers) and employ carefully designed exercise protocols to delineate direct effects on the tumor microenvironment, rather than relying solely on surrogate measures of systemic cardiorespiratory fitness. Key efficacy endpoints should include pathological complete response (pCR) and objective response rate (ORR), complemented by secondary outcomes such as disease-free survival (DFS), chemotherapy-related adverse event rates, and patient quality of life, to establish quantitative links between vascular normalization effects and clinical outcomes. The development and validation of biomarkers reflecting exercise-induced changes in tumor vasculature-such as perfusion parameters, hypoxia markers, and endothelial function indicators-will be essential for mechanistic insights and patient stratification. Recommendations from the American College of Sports Medicine (ACSM) and the “Exercise is Medicine in Oncology” initiative emphasize the importance of individualized exercise prescriptions that account for cancer type, treatment stage, comorbidities, and physical function. Future research should explore tailored exercise regimens for different tumor types and treatment protocols. For instance, a “dose-escalation” approach based on patients' baseline cardiorespiratory fitness could help identify the minimum effective dose required to induce vascular normalization.

In summary, aerobic exercise holds promise as a non-pharmacological adjunctive strategy, with preliminary evidence supporting its potential to improve drug delivery by remodeling tumor vascular function. However, translating this potential into reliable clinical strategies requires overcoming challenges related to evidence quality, understanding of inter-tumor heterogeneity, and elucidation of underlying mechanisms. Future research should prioritize high-quality clinical validation and optimization of personalized exercise regimens to advance the field of exercise oncology from concept to clinical practice.
